# Job satisfaction and regulation in the aged care sector: staff perspectives

**DOI:** 10.1186/s12913-023-10472-0

**Published:** 2023-12-15

**Authors:** Emilie Cameron, Natasha Noble, Jamie Bryant, Grace Norton, Viv Allanson OAM, Rob Sanson-Fisher

**Affiliations:** 1 Health Behaviour Research Collaborative, School of Medicine and Public Health, College of Health and Wellbeing, University of Newcastle, Callaghan, NSW 2308 Australia; 2 Priority Research Centre for Health Behaviour, University of Newcastle, Callaghan, NSW 2308 Australia; 3 Hunter Medical Research Institute, Locked Bag 1000, New Lambton, NSW 2305 Australia; 4 Maroba Caring Communities, 58 Edith Street, Waratah, NSW 2298 Australia

**Keywords:** Residential aged care, Job satisfaction, Quality of care, Aged care workforce, Nursing home

## Abstract

**Background:**

The quality of care provided in residential aged care facilities is largely dependent on the job satisfaction of employees and the organisational framework and systems that they provide care in. This study aimed to explore aged care staff perceptions of job satisfaction, regulation of the sector and the Royal Commission into Aged Care Quality and Safety.

**Methods:**

A cross-sectional survey conducted in 2019-early 2020 with staff employed in various roles at residential aged care services in Australia. The study specific survey collected views and experiences about working in the aged care sector as well as information about their role.

**Results:**

A total of 167 aged care staff completed the survey of which 71% worked in a direct care role. Most participants indicated they thought they were doing a worthwhile and important job (98%), were proud to work in the sector (94%) and found the job personally rewarding (94%). However, participants also reported feeling emotionally drained by the work (37%) and fatigued by having to face a day of work (30%). 72% of participants felt the Royal Commission would lead to improvements in the care provided to residents.

**Conclusion:**

Aged care staff have an overall positive feeling towards their work. Additional support including increasing skills to deliver high-quality care, creating a supportive work environment to reduce job stressors and changes to the way the sector is regulated, are likely to lead to improved care.

**Supplementary Information:**

The online version contains supplementary material available at 10.1186/s12913-023-10472-0.

## Background

Population ageing is one of the most significant social changes to face global communities. There is a need for countries to prepare their long-term care systems to address increasing demand [1]. Across Australia, more than one million older people receive aged care services [2]. In 2020-21, there were 830 providers of residential aged care, 939 providers of home care packages, and more than 1,400 organisations funded to deliver Commonwealth Home Support Program services [3]. The aged care sector in Australia is relatively fragmented, made up predominantly of small to medium providers. The sector relies on a diverse workforce of more than 430,000 staff, approximately 75% of which are direct care workers (such as nurses, Personal Care Attendants and allied health) and 25% non-direct care workers (such as Domestic Services, Administration/ Finance and management) according to the most recent Aged Care Workforce Census Report [4].

The Australian aged care sector is currently experiencing a period of significant change on several fronts. For example, there is an increasing emphasis on consumer choice and rising consumer expectations, with many older Australians choosing to “age in place” at home or seeking more personalised services. In addition to this, an increasing number of older people require complex care for diseases associated with ageing, such as dementia and diabetes, as well as palliative and end-of-life care [5]. Many age care regulatory systems are outdated and need to better accommodate these changing consumer needs [6]. The recent 2021 Royal Commission into Aged Care and Safety uncovered many instances of substandard care and abuse within the aged care system, and called for fundamental and systemic aged care reform to ensure that high quality care is provided [7]. The Royal Commission proposed major reforms to ensure better system governance such as improved quality standards, reporting, guidelines and indicators, and changes to the funding model; as well as improved work conditions and workforce capabilities to improve the quality of care provided by the sector [7]. The COVID-19 pandemic further served to highlight significant shortcomings in the aged care sector, related to governing, management and response, particularly within residential aged care [8].

Personal care workers and nurses are the main care providers in residential aged care facilities, [4] and the quality of care provided is largely dependent on these direct care employees and the organisational framework and systems that they provide care in [9, 10]. Evidence commonly pinpoints understaffing as a major reason behind the inability of nurses and carers to deliver quality care [11]. Staff turnover in aged care is high, [12] with many leaving or planning to leave the sector following the COVID-19 pandemic [13]. High staff turnover rates are associated with negative outcomes such as disruptive behaviour among residents [14]. Job satisfaction among workers within residential aged care facilities is closely associated with staff retention, the quality of care provided to residents and patient outcomes including satisfaction and mortality [15, 16]. Moreover, job satisfaction in residential aged care facilities is particularly important as residents have a long term relationship with staff and are particularly vulnerable to poor quality care [15, 17].

Australian research on job satisfaction among residential aged care workers is relatively limited, and findings on levels of satisfaction are mixed [12, 15, 18–20]. Most previous studies have focused on direct care staff only however, examining job satisfaction among non-direct care staff can help to understand the workplace culture, identify discrepancies that exist between roles, and help identify targeted improvements that may enhance the overall functioning and effectiveness of the entire institution, not just the direct care services [21]. In the context of the Royal Commission calling for fundamental changes to aged care system management, regulation and governance, it is also an important time to examine staff perceptions regarding current regulation and review of the sector, and the impact of the Royal Commission findings. Hence the aims of this study were to explore, among a sample of staff employed in various roles in residential aged care services in Australia, views on job satisfaction, regulation, and the impact of the Royal Commission on the aged care sector.

## Methods

### Design and setting

A cross sectional survey was conducted with staff employed at residential aged care facilities operating in Australia. Data was collected prior to start of the COVID-19 pandemic in Australia between November 2019 and March 2020.

### Residential aged care facility eligibility and recruitment

Any service providing residential care to older people was eligible to participate. Convenience sampling was used with residential aged care facilities known to members of the research team. Residential aged care facilities were approached via email and asked to consider participation in a workforce survey. Facilities who agreed to participate signed a consent form and were assured that collected data would not identify any organisation or individual staff member.

### Participant eligibility

Eligible participants were aged 18 years or older, currently employed in a paid position at a participating aged care service (irrespective of role), and able to read and write English. As the survey required participants to reflect on care provided at the service, individuals who had been employed by the aged care service for less than 3 months were ineligible to participate.

### Participant Recruitment

Two strategies were employed to invite participation, depending on facility preferences:

1. An invitation email was sent to eligible employees from the CEO of the aged care service or an appropriate delegate. Emailed invitations included an invitation letter from the CEO, the participant information sheet, a link to the online version of the survey and information about where to access a hard copy study pack if they preferred to complete the survey via pen-and-paper. A blanket reminder email was sent from the CEO to all potential participants between 2 and 4 weeks after the initial study invitation. Participants were instructed to disregard the email if they had already completed the survey or had chosen not to participate.
2. Hard copy study packs were distributed by administrative and/or management staff of the aged care service. This included distributing packs in staff pigeonholes, at training days or team meetings, at the commencement of shifts, or in staff rooms. Study packs included an invitation letter from the CEO a link to complete the survey online if preferred, the participant information sheet, a hard copy of the survey, and an opaque sealable envelope. Participants could return their completed survey either to a secure drop box placed in a common room at the aged care service, or directly to the research team via reply paid envelope.

### Survey development

The survey (See additional file 1) was designed to explore the views and experiences of staff working in the aged care sector. The survey instrument was developed by a working group that included two behavioural scientists, a palliative care nurse practitioner, the CEO of a residential aged care facility, the director of nursing of a residential aged care facility, a Senior Staff Specialist in Emergency Medicine and Clinical Governance, and a clinical nurse consultant. Item development was based on literature review, including a review of research that has explored the perceptions of aged care workers qualitatively [22–24]. Once developed, survey items were piloted by nine staff at one residential aged care service and items revised based on feedback. The final survey included questions about job satisfaction, and regulation and review outcomes in the industry.

### Data collection

The one-off survey took approximately 15 min to complete and could be completed either online or by pen-and-paper. The survey was anonymous. Participants were asked to complete and return the survey within two weeks of receiving the study invitation. Completion and return of the survey was taken as implied consent to participate in the study.

### Data analysis

Analyses were conducted in Stata 14 [25]. Characteristics of participants are reported as mean (standard deviation) for continuous variables and number (proportion) for categorical variables. For items related to job satisfaction, and regulation those responding agree or strongly agree are presented as endorsing the item. Questions about how often respondents experience the listed feeling about the work they do are reported as frequently (*Every day* and *A few times a week*), occasionally (*Once a week*; *A few times a month*; *Once a month or less*) and rarely or never (A *few times a year*; and *Never)*. As the amount of missing data was low (less than 10%) a complete case analysis was taken for each item. Chi-squared tests were conducted to compare the responses of those in direct and non-direct care roles.

## Results

### Residential aged care facilities

A total of 8 private residential aged care providers agreed to participate. These providers operate a total of 24 facilities and were located in metropolitan and regional areas across New South Wales, Australian Capital Territory, Victoria and Western Australia. There were 5 small facilities (less than 60 beds), 13 medium sized facilities (60–100 beds) and 6 large facilities (over 100 beds). Together the providers employed 2751 staff eligible for the study.

### Participants

A total of 167 aged care staff completed the survey. Table 1 shows the characteristics of the participants. Most participants were female (90%; n = 129), aged over 50 (mean = 51 years; SD = 12.5) and born in Australia (77%, n = 113). Most were employed part time (61%; n = 88) and had worked in aged care for over 10 years (mean = 13 years; SD = 10.2). The majority (71%; n = 104) of participants worked in a direct care role.

**Table 1:** Participant characteristics (N = 167^a^)

	mean (sd)
Age in years	51 (12.5)
Years worked at current service	9 (7.7)
Years worked in aged care	13 (10.2)
Hours worked per week	36 (14.6)
	**N (%)**
Sex	
Female	129 (90%)
Male	15 (10%)
Country of birth	
Australia	113 (77%)
UK	10 (7%)
Other	23 (16%)
Aboriginal or Torres Strait Islander	
Yes	12 (8%)
No	135 (92%)
Education	
High school	26 (18%)
Certificate I, II, III or IV	46 (32%)
Diploma	33 (23%)
Bachelor degree	30 (21%)
Post-graduate degree	11 (7.5%)
Role – direct care	
Personal Care Attendant	58 (39%)
Nurse Practitioner/Registered Nurse	24 (16%)
Enrolled Nurse	13 (8.8%)
Allied health	9 (6.1%)
Role- Non-direct care	
Domestic Services	11 (7.5%)
Administration/ Finance	19 (13%)
Management	5 (3.4%)
Other	8 (5.4%)
Current employment	
Full time	41 (28%)
Part time	88 (61%)
Casual	15 (10%)

^a^ Denominator may not be n = 167 due to missing data.

### Job satisfaction

Agreement with statements about job satisfaction are shown in Table 2. Most participants had made a deliberate choice to work in aged care (79%; n = 132), especially direct care workers (87% compared to 61% for non-direct, Chi^2^ = 12.13, *P* < 0.001), and saw it as a long term job (87%; n = 146). Almost all participants thought they were doing a worthwhile and important job (98%; n = 164), were proud to work in the sector (94%; n = 156) and found the job personally rewarding (94%; n = 156). However, 15% (n = 25) did not feel proud of how residents were cared for at the facility, 17% (n = 28) did not think that the staff at the facility who had direct contact with residents had the right personal attributes to deliver high quality care and 18% (n = 29) did not think staff had the right skills to deliver high-quality care.

**Table 2:** Proportion of participants agreeing with statement about job satisfaction (N = 167)

Item	Total^a^ N (%)	Direct care role ^b^ N (%)	Non-direct care role N (%)	Chi^2^	P-value
I am doing a worthwhile and important job	164 (98%)	105 (99%)	39 (95%)	2.29	0.13
The community has higher expectations about the standard of care that should be provided in residential facilities than ever before	158 (95%)	99 (93%)	40 (98%)	1	0.32
My job is personally rewarding	156 (94%)	97 (92%)	41 (100%)	3.3	0.07
I am proud to work in the aged care sector	156 (94%)	98 (93%)	40 (98%)	1.02	0.31
Working in aged care is a long term job for me	146 (87%)	91 (86%)	39 (95%)	2.49	0.12
I am proud of how residents are cared for at my facility	140 (85%)	88 (84%)	36 (90%)	0.9	0.34
Staff at my facility who have direct contact with residents have the right personal attributes to deliver high quality care	137 (83%)	86 (83%)	33 (80%)	0.1	0.76
Staff at my facility who have direct contact with residents have the right skills to deliver high-quality care	135 (82%)	82 (80%)	34 (83%)	0.21	0.65
The work I do is valued by the community	132 (80%)	79 (75%)	35 (85%)	1.77	0.18
I made a deliberate choice to work in aged care	132 (79%)	92 (87%)	25 (61%)	12.13	< 0.001***

^a^ Denominator may not be n = 167 due to missing data.

^b^ Direct and non-direct care roles do not sum to total due to those missing role information.

Participant’s feelings about their work are shown in Table 3. Most participants reported they frequently deal effectively with the problems of their residents (90%; n = 145) and felt they positively influence other people’s lives (79%; n = 130). However, over a quarter of participants frequently feel emotionally drained by the work (37%; n = 60) and fatigued at the thought of another day of work (30%; n = 50). Direct care workers were significantly more likely to report frequently feeling a strain from working with people all day than non-direct care workers (20% and 2.7% respectively, Chi^2^ = 6.62; *P* = 0.036).

**Table 3:** Frequency at which participants report feeling this way about the work they do (N = 167)

Item	Total^a^ N (%)	Direct care role^b^ N (%)	Non-direct care role N (%)	Chi^2^	P-value
I deal very effectively with the problems of my residents					
Frequently	145 (90%)	100 (92%)	31 (86%)	6.15	0.046*
Occasionally	13 (8%)	9 (8.3%)	3 (8.3%)		
Rarely or Never	4 (2%)	0 (0%)	2 (5.6%)		
I feel the way I treat some residents may appear impersonal					
Frequently	16 (10%)	13 (12%)	2 (5.6%)	2.42	0.30
Occasionally	25 (16%)	19 (18%)	4 (11%)		
Rarely or Never	119 (74%)	76 (70%)	30 (83%)		
I feel emotionally drained from my work					
Frequently	60 (37%)	42 (38%)	11 (30%)	5.19	0.075
Occasionally	69 (42%)	49 (45%)	13 (35%)		
Rarely or Never	35 (21%)	19 (17%)	13 (35%)		
I feel fatigued when I get up in the morning and have to face another day on the job					
Frequently	50 (30%)	33 (30%)	11 (30%)	3.95	0.14
Occasionally	55 (34%)	41 (37%)	8 (22%)		
Rarely or Never	59 (36%)	36 (33%)	18 (49%)		
I have less empathy towards people since I took this job					
Frequently	10 (6%)	7 (6.4%)	2 (5.6%)	3.22	0.20
Occasionally	23 (14%)	19 (17%)	2 (5.6%)		
Rarely or Never	129 (80%)	83 (76%)	32 (89%)		
I feel I’m positively influencing other people’s lives through my work					
Frequently	130 (79%)	94 (85%)	23 (62%)	12.08	0.002**
Occasionally	16 (10%)	6 (5.5%)	9 (24%)		
Rarely or Never	18 (11%)	10 (9.1%)	5 (14%)		
Working with people all day is really a strain for me					
Frequently	26 (16%)	22 (20%)	1 (2.7%)	6.62	0.036*
Occasionally	42 (26%)	29 (26%)	10 (27%)		
Rarely or Never	96 (59%)	59 (54%)	26 (70%)		
I don’t really care what happens to some residents					
Frequently	3 (2%)	2 (1.8%)	1 (2.7%)	1.13	0.57
Occasionally	4 (2%)	3 (2.7%)	0 (0%)		
Rarely or Never	157 (96%)	105 (95%)	36 (97%)		
I feel exhilarated after working closely with my residents					
Frequently	98 (62%)	71 (66%)	20 (59%)	1.08	0.58
Occasionally	37 (24%)	24 (22%)	8 (24%)		
Rarely or Never	22 (14%)	12 (11%)	6 (18%)		

^a^ Denominator may not be n = 167 due to missing data.

^b^ Direct and non-direct care roles do not sum to total due to those missing role information.

### Regulation and review

Table 4 shows participant agreement with statements relating to regulation and review in the aged care sector. Many felt that the regulatory bureaucracies were distracting from providing high level care to residents (88%; n = 129) and there was a tick box culture (80%; n = 113). In regard to the Royal Commission, nearly three quarters (72%; n = 105) felt that it will result in improvements to the care provided to residents. However, they also felt that it was contributing to families feeling uncomfortable about using aged care (78%; n = 116) and almost a third (29%; n = 43) reported that it made them ashamed to work in aged care. This was particularly felt by direct care workers (35% compared to 15% for non-direct; Chi^2^ = 6.02; *P* = 0.01). Only 55% (n = 80) felt that the current regulatory framework functions adequately to protect older people. Interestingly, 76% (n = 77) of those in a direct care role thought that poorly performing services should be managed out of the system, compared to just 46% (n = 19) of those in a non-direct role (Chi^2^ = 11.9; *P* < 0.001).

**Table 4:** Proportion of agreement with statements about regulation and review in the aged care sector (N = 148)

Item	Total^a^ N (%)	Direct Care role^b^ N (%)	Non-direct Care role N (%)	Chi^2^	P-value
Current regulatory bureaucracies distract from providing high quality care to residents	129 (88%)	91 (88%)	36 (90%)	0.17	0.68
The Royal Commission has contributed to families feeling uncomfortable about using aged care	116 (78%)	84 (80%)	30 (73%)	0.8	0.37
There is a tick-box culture of compliance in the aged care sector	113 (80%)	82 (82%)	30 (77%)	0.46	0.5
The Royal Commission will result in improvements to the care provided to residents	105 (72%)	74 (71%)	29 (73%)	0.03	0.87
The safety and well-being of residents is the core principle and focus of the current regulatory system	105 (74%)	76 (75%)	28 (72%)	0.18	0.68
Poorly performing aged care service providers should be managed out of the system, not managed back to compliance	98 (68%)	77 (76%)	19 (46%)	11.9	< 0.001***
Much of the current reporting does not make a difference to the delivery of care quality	96 (67%)	67 (66%)	28 (70%)	0.24	0.62
Creating new rules about how care should be provided will improve the quality of care residents receive	91 (62%)	68 (65%)	22 (55%)	1.17	0.28
The current regulatory framework functions adequately to protect older people	80 (55%)	60 (58%)	19 (48%)	1.21	0.27
Because of the Royal Commission, I have had to justify my work to other people in social situations	60 (41%)	47 (45%)	12 (31%)	2.3	0.13
Because of the Royal Commission, I feel ashamed to tell people I work in aged care	43 (29%)	37 (35%)	6 (15%)	6.02	0.01*

^a^ Denominator may not be n = 148 due to missing data.

^b^ Direct and non-direct care roles do not sum to total due to those missing role information.

## Discussion

Our study found that overall job satisfaction among residential aged care staff was high, with over 90% agreeing with statements such as finding their work worthwhile and important, finding the job personally rewarding, and being proud to work in the sector. Direct care workers, in particular, had made a deliberate choice to work in the aged care sector. High overall job satisfaction is consistent with most previous studies in the Australian context, [12, 20] although Healy et al. reported that female residential aged care workers were twice as likely as the general female Australian workforce to be dissatisfied with their jobs [18]. Despite overall high satisfaction, there were some aspects of work that staff in the current sample were less satisfied with. For example, 15% of respondents were not proud of the care residents at their facility were receiving, and almost one in five respondents (18%) felt that staff at the facility did not have the right skills to deliver high-quality care. The latter finding confirms well established calls for better staffing levels and skills mix in residential aged care [26]. Our findings also echo previous qualitative work suggesting that staff perceptions of the rewarding aspects of residential aged care work serve to counterbalance some of the challenges of such work [23].

While the majority of staff cared what happened to the residents and felt that they were positively influencing other people’s lives through their work, some staff reported negative feelings associated with work. For example, more than one in three staff (37%) reported frequently feeling emotionally drained from their work, and over a quarter (30%) frequently felt fatigued at the thought of another day on the job. This was especially the case for direct care workers, who were more likely than non-direct workers to report feeling that working with people all day was a strain. Such findings are important in terms of retaining the aged care work force, a sector which struggles to attract skilled workers and experiences high staff attrition rates [27]. The recent COVID-19 pandemic had a profound negative impact on residential aged care workers’ distress and mental health, [28] and has seen many staff leaving the sector [13]. Addressing issues with the work environment, such as heavy workload, psychological and emotional stress, poor organizational support, and lack of education and training, is critical for ensuring an adequate aged care workforce into the future [14, 23, 29]. Such support could include training in adaptive coping skills and to improve confidence, factors which have been previously identified for preventing occupational burnout for Residential aged care staff in Australia [30]. In addition, organisational support could emphasise the rewards experienced by many direct care workers, such as feeling effective in dealing with residents problems and positively influencing residents lives.

Most participants agreed that regulatory bureaucracies tend to distract from providing high quality care to residents and that there tends to be a tick-box culture of compliance in the aged care sector. Respondents also indicated that current regulation and review of the sector is not effective, with over two-thirds (67%) agreeing that the current reporting does not make a difference to quality of care, and 45% of respondents indicating that the current regulatory framework does not function adequately to protect older people. Along with factors such as irregular schedules and high workloads, regulatory requirements have been identified as contributing to the emotional strain of aged care workers [31]. The Royal Commission also noted that current regulatory arrangements are failing to provide an acceptable and reliable aged care system [7]. In line with the findings of this study, the Royal Commission recommended that a new Act governing the aged care system is needed, one which is person centred and which puts the needs and preferences of older people first [7]. Interestingly, staff’s perceptions regarding the Royal Commission were somewhat mixed. While almost three quarters (72%) felt that it will result in improvements to the care provided to residents, a large majority of staff also felt that it was contributing to families feeling uncomfortable about using aged care (78%) and almost a third (29%) reported that it made them ashamed to work in aged care. The latter was especially evident for direct care workers. While the Royal Commission recommended urgent reform of the aged care system, care needs to be taken to ensure that the changes do not increase the complexity of aged care roles and detract from their value [32] whilst also ensuring reform is effective and does not create an increased tick-box culture of compliance. Given that valuing aged care work and retaining staff are fundamental to providing quality residential aged care, there is a need more broadly to increase public awareness of the importance and value of aged care work, and to promote and advocate for the positive aspects of aged care work [23].

Workplace culture, defined as the shared beliefs, values, attitudes and behaviours that characterize the work environment, has been shown to influence patient outcomes in aged care settings [21]. The lack of discrepancy in levels of job satisfaction and feelings towards the workplace found in this study between direct and non-direct care workers suggests a positive workplace culture. However, some differences were apparent in the level of strain felt by direct care staff from working with people all day and a feeling of shame about the work they perform due to the royal commission. Direct care workers also felt more strongly that they were positively influencing other’s lives through their work and had made a deliberate choice to work in aged care.

It should be noted that the survey was conducted shortly prior to the outbreak of the COVID-19 pandemic in Australia. The COVID-19 pandemic disproportionately affected both consumers and the workforce in the aged care sector, [4] with residential aged care workers faced with increased workloads due to reduced staff availability, supporting residents isolated from families, worry about spreading the virus to residents, and witnessing COVID-19 deaths of residents [28]. Such factors are likely to have impacted on views around job satisfaction and perceptions of regulation of the sector.

### Limitations

This study had a relatively small sample size and was drawn from a sample of eight privately managed aged care facilities. Therefore, the findings may not be generalisable to the entire aged care work force nationally, particularly staff working in facilities operated by government or not-for-profit organisations. Motivated staff are more likely to have responded to the survey, so the findings may reflect more positive views of working in the aged care sector compared to those of the broader workforce. Those with less positive views may have been reluctant to participate, despite the assurance of anonymity. In addition, the survey was developed specifically for the study. Although developed in collaboration with an expert working group, it may not have captured all key aspects or issues associated with working in the aged care sector. For example, our survey did not assess factors such as satisfaction with work schedules or pay rates.

## Conclusions

Staff working in residential aged care facilities have overall positive feelings about working in the aged care sector and high job satisfaction. However, there are some aspects of work where aged care staff may need additional support, such as increasing skills to deliver high-quality care, and creating supportive work environments which reduce job stressors and nurture the perceived rewards associated with working in the sector especially among direct care workers. Results also indicate the need for a change in the regulatory approach to the aged care sector, with many staff agreeing that current approaches are not meeting the needs of residents. It remains to be seen how implementation of the major regulatory changes recommended by the Royal Commission impacts upon the care of residents and the job satisfaction and wellbeing of residential aged care workers.

### Electronic supplementary material

Below is the link to the electronic supplementary material.

Supplementary Material 1

## Data Availability

The datasets generated during during the current study are available from the corresponding author on reasonable request.
