# Supplementary material for: Job satisfaction and regulation in the aged care sector: staff perspectives
Source: BMC Health Serv Res. 2023 Dec 15;23:1421. doi: 10.1186/s12913-023-10472-0 (PMC10722849; doi:10.1186/s12913-023-10472-0)
Supplement: Supplementary file 1 — Supplementary Material 1 [file 12913_2023_10472_MOESM1_ESM.docx]

**Additional File 1: *Aged Care Workforce Survey***

**How you feel about your job**

- The following questions ask about how you feel about working in the aged care sector.
- Please indicate how strongly you agree or disagree with each of the following statements by circling the corresponding number.

|  | **Strongly**  **disagree** | **Disagree** | **Agree** | **Strongly**  **agree** |
| --- | --- | --- | --- | --- |
| 1. I am doing a worthwhile and important job |  |  |  |  |
| 1. My job is personally rewarding |  |  |  |  |
| 1. I am proud to work in the aged care sector |  |  |  |  |
| 1. I am proud of how residents are cared for at my facility |  |  |  |  |
| 1. Staff at my facility who have direct contact with residents have the right skills to deliver high-quality care |  |  |  |  |
| 1. Staff at my facility who have direct contact with residents have the right personal attributes to deliver high quality care |  |  |  |  |
| 1. The work I do is valued by the community |  |  |  |  |
| 1. The community has higher expectations about the standard of care that should be provided in residential facilities than ever before |  |  |  |  |
| 1. Working in aged care is a long term job for me |  |  |  |  |
| 1. I made a deliberate choice to work in aged care |  |  |  |  |

- The following questions ask how you feel about the work you do.
- For each question, please indicate how often you feel like this by circling the corresponding number.

|  | **Every day** | **A few times a week** | **Once a week** | **A few times a month** | **Once a month or less** | **A few times a year** | **Never** |
| --- | --- | --- | --- | --- | --- | --- | --- |
| 1. I deal very effectively with the problems of my residents |  |  |  |  |  |  |  |
| 1. I feel the way I treat some residents may appear impersonal |  |  |  |  |  |  |  |
| 1. I feel emotionally drained from my work |  |  |  |  |  |  |  |
| 1. I feel fatigued when I get up in the morning and have to face another day on the job |  |  |  |  |  |  |  |
| 1. I have less empathy towards people since I took this job |  |  |  |  |  |  |  |
| 1. I feel I'm positively influencing other people's lives through my work |  |  |  |  |  |  |  |
| 1. Working with people all day is really a strain for me |  |  |  |  |  |  |  |
| 1. I don't really care what happens to some residents |  |  |  |  |  |  |  |
| 1. I feel exhilarated after working closely with my residents |  |  |  |  |  |  |  |

**Regulation and review of aged care quality and safety**

- The following questions ask for your views about regulation in the aged care sector, as well as about the current Royal Commission into Aged Care Quality and Safety.
- Please indicate how strongly you agree or disagree with each of the following statements by circling the corresponding number.

|  | **Strongly**  **disagree** | **Disagree** | **Agree** | **Strongly**  **agree** |
| --- | --- | --- | --- | --- |
| 1. The current regulatory framework functions adequately to protect older people |  |  |  |  |
| 1. Much of the current reporting does not make a difference to the delivery of care quality |  |  |  |  |
| 1. Creating new rules about how care should be provided will improve the quality of care residents receive |  |  |  |  |
| 1. Current regulatory bureaucracies distract from providing high quality care to residents |  |  |  |  |
| 1. There is a tick-box culture of compliance in the aged care sector |  |  |  |  |
| 1. Poorly performing aged care service providers should be managed out of the system, not managed back to compliance |  |  |  |  |
| 1. The safety and well-being of residents is the core principle and focus of the current regulatory system |  |  |  |  |
| 1. The Royal Commission will result in improvements to the care provided to residents |  |  |  |  |
| 1. Because of the Royal Commission, I feel ashamed to tell people I work in aged care |  |  |  |  |
| 1. The Royal Commission has contributed to families feeling uncomfortable about using aged care |  |  |  |  |
| 1. Because of the Royal Commission, I have had to justify my work to other people in social situations |  |  |  |  |
